# A Variant PfCRT Isoform Can Contribute to *Plasmodium falciparum* Resistance to the First-Line Partner Drug Piperaquine

**DOI:** 10.1128/mBio.00303-17

**Published:** 2017-05-09

**Authors:** Satish K. Dhingra, Devasha Redhi, Jill M. Combrinck, Tomas Yeo, John Okombo, Philipp P. Henrich, Annie N. Cowell, Purva Gupta, Matthew L. Stegman, Jonathan M. Hoke, Roland A. Cooper, Elizabeth Winzeler, Sachel Mok, Timothy J. Egan, David A. Fidock

**Affiliations:** a Department of Microbiology and Immunology, Columbia University Medical Center, New York, New York, USA; b Division of Pharmacology, Department of Medicine, University of Cape Town, Cape Town, South Africa; c Department of Chemistry, University of Cape Town, Cape Town, South Africa; d Division of Infectious Diseases, Department of Internal Medicine, University of California, San Diego, California, USA; e Division of Host-Microbe Systems & Therapeutics, Department of Pediatrics, University of California, San Diego, California, USA; f Department of Natural Sciences and Mathematics, Dominican University of California, San Rafael, California, USA; g Department of Biological Sciences, Old Dominion University, Norfolk, Virginia, USA; h Division of Infectious Diseases, Department of Medicine, Columbia University Medical Center, New York, New York, USA; National Institutes of Health

**Keywords:** malaria, PfCRT, *Plasmodium falciparum*, artemisinin-based combination therapies, digestive vacuole, genome editing, heme detoxification, piperaquine resistance

## Abstract

Current efforts to reduce the global burden of malaria are threatened by the rapid spread throughout Asia of *Plasmodium falciparum* resistance to artemisinin-based combination therapies, which includes increasing rates of clinical failure with dihydroartemisinin plus piperaquine (PPQ) in Cambodia. Using zinc finger nuclease-based gene editing, we report that addition of the C101F mutation to the chloroquine (CQ) resistance-conferring PfCRT Dd2 isoform common to Asia can confer PPQ resistance to cultured parasites. Resistance was demonstrated as significantly higher PPQ concentrations causing 90% inhibition of parasite growth (IC_90_) or 50% parasite killing (50% lethal dose [LD_50_]). This mutation also reversed Dd2-mediated CQ resistance, sensitized parasites to amodiaquine, quinine, and artemisinin, and conferred amantadine and blasticidin resistance. Using heme fractionation assays, we demonstrate that PPQ causes a buildup of reactive free heme and inhibits the formation of chemically inert hemozoin crystals. Our data evoke inhibition of heme detoxification in the parasite’s acidic digestive vacuole as the primary mode of both the *bis*-aminoquinoline PPQ and the related 4-aminoquinoline CQ. Both drugs also inhibit hemoglobin proteolysis at elevated concentrations, suggesting an additional mode of action. Isogenic lines differing in their *pfmdr1* copy number showed equivalent PPQ susceptibilities. We propose that mutations in PfCRT could contribute to a multifactorial basis of PPQ resistance in field isolates.

## IMPORTANCE

The global agenda to eliminate malaria depends on the continued success of artemisinin-based combination therapies (ACTs), which target the asexual blood stages of the intracellular parasite *Plasmodium*. Partial resistance to artemisinin, however, is now established in Southeast Asia, exposing the partner drugs to increased selective pressure. *Plasmodium falciparum* resistance to the first-line partner piperaquine (PPQ) is now spreading rapidly in Cambodia, resulting in clinical treatment failures. Here, we report that a variant form of the *Plasmodium falciparum* chloroquine resistance transporter, harboring a C101F mutation edited into the chloroquine (CQ)-resistant Dd2 isoform prevalent in Asia, can confer PPQ resistance in cultured parasites. This was accompanied by a loss of CQ resistance. Biochemical assays showed that PPQ, like CQ, inhibits the detoxification of reactive heme that is formed by parasite-mediated catabolism of host hemoglobin. We propose that novel PfCRT variants emerging in the field could contribute to a multigenic basis of PPQ resistance.

## INTRODUCTION

Malaria remains a leading cause for mortality and morbidity worldwide, responsible for an estimated 429,000 deaths and 212 million cases in 2015. Over 90% of these deaths occurred in sub-Saharan Africa, primarily among children below the age of 5 (1). The clinical treatment of malaria relies almost exclusively on the use of artemisinin (ART)-based combination therapies (ACTs) (2). These combine a fast-acting, rapidly eliminated ART derivative with one slower-acting partner drug with a longer half-life. The current repertoire of partner drugs includes piperaquine (PPQ), amodiaquine (ADQ), lumefantrine (LMF), and mefloquine (MFQ) (see Fig. S1 in the supplemental material). ACTs, designed to suppress the emergence of multidrug resistance, have proven to be an effective treatment of *Plasmodium falciparum* infections resistant to the former first-line drugs chloroquine (CQ) and sulfadoxine-pyrimethamine. Their impressive efficacy has been a major contributor to the 2-fold reduction in global malaria mortality achieved over the past decade, leading to calls for a global malaria elimination campaign predicated on the sustained efficacy of ACTs and mosquito vector control programs (3).

10.1128/mBio.00303-17.1 FIG S1  Chemical structures of different antimalarials. DHA, dihydroartemisinin. Download FIG S1, TIF file, 0.6 MB. Copyright © 2017 Dhingra et al. 2017 Dhingra et al. This content is distributed under the terms of the Creative Commons Attribution 4.0 International license.

Emerging resistance to ACT drugs, however, now threatens to reverse these recent gains in malaria control. Resistance to the core ART derivatives, which manifests as delayed parasite clearance following treatment with artesunate (AS) monotherapy or an ACT, is now established in many areas of Southeast Asia (4–9). This scenario recalls the earlier situation where resistance to CQ and sulfadoxine-pyrimethamine first arose in Asia before spreading to Africa, to devastating effect (10). *In vitro* selection studies and whole-genome sequence analysis of ART-resistant or -sensitive field isolates and drug-pressured parasite lines have associated ART resistance with point mutations in Kelch13 (K13) (11). A primary role for these K13 mutations was recently confirmed using zinc finger nuclease (ZFN)-based *k13* gene editing (12, 13). Recent epidemiological studies confirm a strong association between certain point mutations in K13 and clinically defined ART resistance (14–19). To date, K13 mutations are very rare and of no known clinical impact in Africa (15, 20, 21), where rates of transmission, occurrence of mixed infections, and levels of host immunity are generally much higher and where infections are less often treated compared to Asia (22). The predominant Asian K13 mutation, C580Y, however, has emerged in the low-transmission setting of French Guiana (23), suggesting that ART resistance might soon take hold in South America. Resistance to ARTs results in a greater selective pressure on the accompanying partner drug, increasing the probability that multidrug resistance will emerge and cause clinical treatment failures.

PPQ combined with the active ART metabolite dihydroartemisinin (DHA) is currently the first-line antimalarial drug in Cambodia and several neighboring countries, where it initially proved highly effective as a replacement for the failing drug combination of AS+MFQ (24). Mathematical modeling of clinical and epidemiological data from Africa suggests that DHA+PPQ has a longer posttreatment prophylactic period and is more likely to reduce malaria transmission than artemether (ATM)+LMF, which is the most widely used ACT in Africa (25). Despite its extensive clinical use, the mode of action of PPQ has received relatively little attention. Its *bis*-quinoline structure, comprised of two CQ-like 4-aminoquinoline moieties with a central linker, has led to the premise that PPQ, like CQ, acts via inhibition of heme detoxification, a critical process for *P. falciparum* blood-stage survival (26–28). Heme detoxification occurs in the blood-stage parasite’s digestive vacuole (DV), a highly acidic (pH 5.2 to 5.5) compartment wherein a suite of aspartic and cysteine proteases degrade hemoglobin (Hb) and where the released reactive heme is detoxified via its incorporation as β-hematin dimers into chemically inert hemozoin (Hz) crystals (29–35).

Consistent with a role in inhibiting heme detoxification, both PPQ and CQ are weak bases that accumulate up to 1,000-fold in the DV (36–38). *P. falciparum* resistance to CQ is mediated primarily by PfCRT, a transmembrane protein present on the DV membrane whose mutant isoforms appear to efflux this drug out of the DV (39–42). These isoforms, which include the Dd2 haplotype dominant in Asia, earlier spread across the globe under a CQ selective sweep (43). Recent data confirm a secondary contribution to CQ resistance by some mutant isoforms of PfMDR1, an ABC transporter that also resides on the DV membrane (44–47). Both PfCRT and PfMDR1 are known to affect *P. falciparum* susceptibility to multiple other antimalarials, including ADQ, LMF, and ART (48, 49).

Reports now document that PPQ resistance has emerged in Cambodia and is spreading at an alarming rate, accompanied by increasing rates of DHA+PPQ treatment failure (50–55). This recent turn of events creates a precarious scenario of multidrug-resistant malaria that could become untreatable unless alternative treatment strategies are rapidly implemented (56, 57). Identification of molecular determinants of PPQ resistance can accelerate the implementation of markers to track its regional emergence and spread. Until very recently, however, efforts to define PPQ resistance loci have been hindered by the lack of available resistant isolates or parasite lines and a phenotype that has been difficult to quantify, particularly as PPQ 50% inhibitory concentrations (IC_50_s) of resistant parasites have been reported to be unreliable and do not necessarily capture differences between parasites in their dose-response profiles and rates of parasite survival following PPQ pressure (58, 59). Genome-wide association studies have very recently reported an association with an increase in the copy number of plasmepsins 2 and 3, which are aspartic proteases that participate in Hb digestion (54, 55). These studies did not detect an association with *pfcrt*, although that analysis was potentially complicated by the notoriously difficult sequence coverage across the 13-exon *pfcrt* locus and by the existence of multiple rare variants (including H97Y, M343L, and G353V) that would need to be both individually and collectively examined. Of note, a recent study identified those PfCRT variants only in parasites that were PPQ resistant *in vitro* (59).

Several years ago, we reported the *in vitro* selection of PPQ-resistant Dd2 lines (38). Whole-genome tiling array analysis and sequence confirmation of genetic changes identified a novel C101F mutation in PfCRT. Tiling array analysis also suggested the deamplification of an 82-kb region on chromosome 5 that encompasses *pfmdr1* and a possible amplification event of an adjacent 63-kb region. Some lines were reported to revert to a sensitive phenotype upon continuous culturing without PPQ pressure, based on IC_50_s. These revertant parasites appeared to have further deamplified the 82-kb region and lost the 63-kb amplification on chromosome 5. However, these revertant lines still carried the PfCRT^C101F^ mutation. Our closer inspection of the revertant parasites identified a shift in the PPQ IC_90_ value, indicating a possible contribution of the PfCRT^C101F^ polymorphism to the PPQ resistance phenotype. Furthermore, more recent whole-genome sequence analysis failed to confirm the earlier tiling array predictions of changes in copy number of the 63-kb region, arguing against its earlier proposed role in resistance. In this study, we further examine the possible contribution of the PfCRT^C101F^ mutation by leveraging recent advances in *pfcrt* gene editing achieved with customized ZFNs (60, 61).

We also report herein our assessment of PPQ susceptibility in isogenic parasites that differed in their *pfmdr1* copy number as a result of targeted gene disruption (62). The rationale for this study came from recent therapeutic efficacy studies with DHA+PPQ in Cambodia that provided evidence of a slight selection advantage for parasites with single-copy *pfmdr1* among recrudescent infections (53) or slightly higher PPQ IC_50_s in the single-copy compared to multicopy *pfmdr1* parasites (51). These studies evoke the possibility that the reduction in *pfmdr1* copy number might have been more a result of Cambodia having earlier abandoned the use of MFQ, which is known to select for multicopy *pfmdr1* (63), as opposed to PPQ itself selecting for single-copy *pfmdr1* parasites. *In vitro* studies with field isolates have also provided conflicting evidence on a possible association with *pfmdr1* copy number (54, 55, 58, 64–67), underlying a need to investigate this using isogenic parasite lines.

## RESULTS

### ZFN-mediated editing to introduce PfCRT C101F into Dd2 parasites.

**FIG 1:**
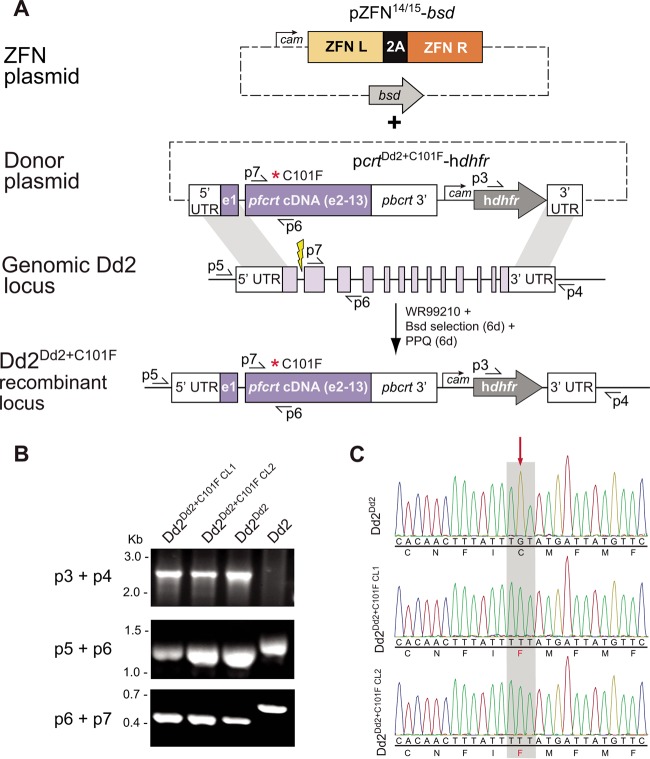
Zinc finger nuclease (ZFN)-mediated editing strategy of Dd2. (A) Schematic of *pfcrt* editing, resulting in introduction of the C101F mutation. Dd2 parasites were transformed with the donor plasmid p*crt*^Dd2+C101F^-h*dhfr*. This plasmid carries the cDNA version of a modified Dd2 *pfcrt* allele harboring the PfCRT^C101F^ mutation as well as a human *dhfr* cassette that mediates resistance to the selection agent WR99210. Episomally enriched Dd2 parasites were then transformed with the ZFN-expressing plasmid pZFN^14/15^-*bsd*. These 2A-linked ZFNs target the *pfcrt* intron 1-exon 2 junction (yellow thunderbolt). Homologous recombination-based repair, triggered by the ZFN-induced double-stranded break, utilized the donor template to generate the recombinant parasites Dd2^Dd2+C101F^. (B) PCR-based screening of *pfcrt*-modified and parental Dd2 parasites. Removal of introns from the *pfcrt* locus of the recombinant Dd2 parasites yielded shorter PCR amplicons compared to parental Dd2. This screen used the primer pairs p3+p4 (Dd2^Dd2+C101F^ and Dd2^Dd2^, 2.5 kb; Dd2, no product), p5+p6 (Dd2^Dd2+C101F^ and Dd2^Dd2^, 1.2 kb; Dd2, 1.4 kb), and p6+p7 (Dd2^Dd2+C101F^ and Dd2^Dd2^, 0.4 kb; Dd2, 0.6 kb). Primer positions are depicted in panel A. (C) Chromatograms of a region of exon 2 obtained from sequencing *pfcrt* cDNA from Dd2^Dd2^ and the two *pfcrt*-modified clones Dd2^Dd2+C101F CL1^ and Dd2^Dd2+C101F CL2^. The red arrow depicts the introduction of the C101F mutation at codon 101 in the edited clones.

We introduced the C101F mutation into the endogenous *pfcrt* locus of Dd2 parasites using ZFN-mediated genome editing (Fig. 1A). These customized ZFNs bind opposite strands of the *pfcrt* intron 1-exon 2 junction, producing a double-stranded break (60). DNA repair proceeded via homologous recombination, as *P. falciparum* lacks the nonhomologous end-joining pathway (68). We leveraged this unique feature to edit *pfcrt* by providing a homologous template carrying the mutation of interest on a donor plasmid.

To generate the edited clones, we electroporated Dd2 parasites with the p*crt*^Dd2^-h*dhfr* donor and pZFN^14/15^-*bsd* ZFN-carrying plasmids that express the human *dhfr* (h*dhfr*) and blasticidin-S deaminase (*bsd*) selectable markers, respectively (Fig. 1A; see Materials and Methods). Our selection regimen also included 6 days of pressure with 40 nM PPQ (corresponding to 1.5× the IC_90_ value in Dd2 parasites). Repeated attempts to achieve editing without short-term PPQ pulsing were consistently unsuccessful, presumably because of the poor growth that precluded expansion of edited lines in the absence of direct selection (see below).

Successfully edited parasites were identified by PCR and sequencing (Fig. 1B and C) and cloned by limiting dilution, yielding Dd2^Dd2+C101F CL1^ and Dd2^Dd2+C101F CL2^ (i.e., Dd2^Dd2+C101F^ clones 1 and 2, respectively) (Table 1). The recombinant control line Dd2^Dd2^, which accounts for the loss of introns from the *pfcrt* gene and the introduction of the human *dhfr* cassette downstream, has been previously described (Table 1) (69).

**TABLE 1:** Haplotypes of *pfcrt*-modified and parental Dd2 parasites

Parasite line^a^	Altered PfCRT haplotype	Donor plasmid	ZFN plasmid	PfCRT haplotype at position^b^:									
				72	74	75	76	101	220	271	326	356	371
Dd2	No	None	None	C	I	E	T	C	S	E	S	T	I
Dd2^Dd2^	No	p*crt*^Dd2^-h*dhfr*	pZFN^14/15^-*bsd*	C	I	E	T	C	S	E	S	T	I
Dd2^Dd2+C101F CL1^	Yes	p*crt*^Dd2+C101F^-h*dhfr*	pZFN^14/15^-*bsd*	C	I	E	T	F	S	E	S	T	I
Dd2^Dd2+C101F CL2^	Yes	p*crt*^Dd2+C101F^-h*dhfr*	pZFN^14/15^-*bsd*	C	I	E	T	F	S	E	S	T	I
3D7	No	None	None	C	M	N	K	C	A	Q	N	I	R

^a^ The name of the transfected strain is shown, followed by a superscript, which refers to the transfected haplotype. 3D7 was added to show the canonical wild-type haplotype observed in chloroquine-sensitive parasites.

^b^ Underlined letters indicate residues that were modified compared to the parental line.

To assess whether the 6 days of treatment with 40 nM PPQ selected for any genetic changes in addition to the introduction of the C101F mutation into *pfcrt*, we performed whole-genome sequence analysis of the two Dd2^Dd2+C101F^ clones and the recombinant control line Dd2^Dd2^. Illumina-based sequencing and analysis, conducted independently by two separate groups, confirmed the introduction of the C101F polymorphism into the *pfcrt* gene in both ZFN-edited Dd2^Dd2+C101F^ clones. We also observed another mutation (R247S) in a gene (PF3D7_0912500) that codes putatively for an SAP domain-containing nucleic acid-binding protein. It would appear unlikely that this gene would contribute to PPQ resistance, and we suspect this is instead an unrelated mutation that arose spontaneously during the culture period. Importantly, no amplifications or deamplifications were observed in either Dd2^Dd2+C101F^ clone, including at the *pfmdr1* locus.

We also carried out control transfection experiments as a separate approach to assess whether our use of short-term PPQ pressure might have selected for resistance independent of the PfCRT^C101F^ mutation. Dd2 and Dd2^Dd2^ parasites were electroporated on two separate occasions with the pZFN^14/15^ plasmid (used in our *pfcrt* gene editing transfections), but not the p*crt*^Dd2^-h*dhfr* donor plasmid used to edit *pfcrt*. We then applied 2 µg/ml of blasticidin (BSD) and 40 nM of PPQ pressure for 6 days. These selection conditions mirrored the ones used to generate the *pfcrt*-modified Dd2^Dd2+C101F^ clones. None of the four transfections became positive within 80 days postelectroporation (compared to the Dd2^Dd2+C101F^ transfections that became positive after 6 weeks), providing evidence that the 40 nM PPQ pulse did not independently select for resistant parasites but instead gave a slight advantage in selecting for Dd2^Dd2+C101F^ parasites that were edited to carry the PfCRT^C101F^ mutation introduced from the donor plasmid.

### PfCRT^C101F^ confers a highly PPQ-resistant phenotype accompanied by distended digestive vacuoles and reduced rate of growth.

**FIG 2:**
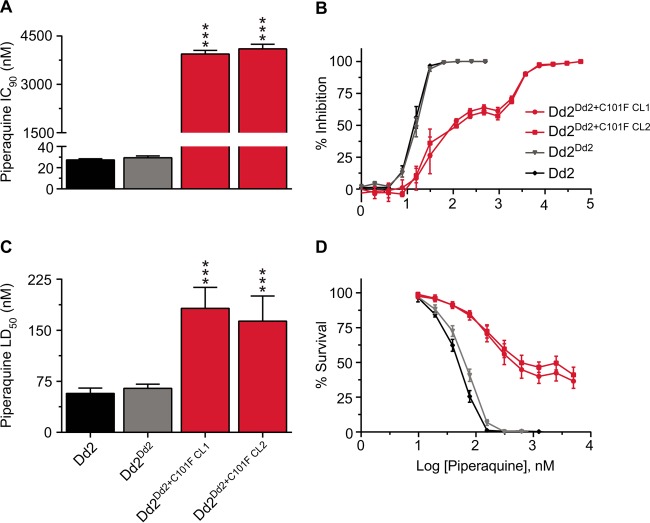
*In vitro* IC_90_ and LD_50_ profiles of the *pfcrt*-modified lines for piperaquine. (A) Piperaquine (PPQ) IC_90_ (nanomolar concentration) profiles of *pfcrt*-modified lines and their Dd2 parent. Parasites were exposed to a range of PPQ concentrations for 72 h, and parasitemias were measured by flow cytometry. Mean ± SEM IC_90_ values are presented for PPQ (assays performed in duplicate on 9 to 11 separate occasions [Table S1]). (B) PPQ concentration-dependent growth inhibition curves (mean ± SEM) for *pfcrt*-modified and parental Dd2 lines. (C) PPQ cytocidal potency against the same lines. LD_50_ values were determined by incubating parasites with a range of PPQ concentrations (2-fold dilution, starting concentration of 5,000 nM) for 6 h, after which the drug was removed. Growth was assessed 48 h later by determining the parasitemia using flow cytometry. LD_50_ values are presented as means ± SEM (assays performed in duplicate on 7 to 9 separate occasions [Table S1]). (D) Parasite survival curves (with means ± SEM) of PPQ-treated parasites tested in cytocidal assays (see Table S2 in the supplemental material for representative parasitemias). For panels A and C, the Dd2^Dd2+C101F^ clones were statistically compared to the isogenic control Dd2^Dd2^ using two-tailed Mann-Whitney *U* tests. ***, *P* < 0.001.

Two clones of Dd2^Dd2+C101F^, the cloned recombinant control line Dd2^Dd2^, and the unmodified parental Dd2 line were assessed for their *in vitro* susceptibilities to a panel of antimalarials. Initial susceptibility assays subjected parasites to a range of PPQ concentrations for 72 h, after which parasites were labeled with SYBR green I and MitoTracker Deep Red and parasitemias determined by flow cytometry. Results showed that both *pfcrt*-modified Dd2^Dd2+C101F^ clones exhibited a highly PPQ-resistant phenotype with an ~140-fold shift in IC_90_ values compared to the Dd2^Dd2^ isogenic recombinant control (mean ± standard error of the mean [SEM] IC_90_ values of 3,942 ± 113 nM and 4,104 ± 142 nM in the two C101F clones compared to a value of 29 ± 2 nM for Dd2^Dd2^; *P* < 0.001) (Fig. 2A; see Table S1 in the supplemental material). We were unable to accurately derive the IC_50_s for these parasites as the dose-response curves showed a bimodal distribution with intermediate levels of inhibition across a range of ~200 nM to 2,000 nM (Fig. 2B) and incomplete killing up to 5,000 nM (see Table S2 in the supplemental material). We could only achieve 100% growth inhibition at extremely high PPQ concentrations (up to 60,000 nM). Atypical dose-response curves have also been observed in PPQ-resistant field isolates from Cambodia (54, 59, 70).

10.1128/mBio.00303-17.6 TABLE S1  Mean ± SEM IC_50_, IC_90_, and LD_50_ values (nanomolar concentrations) of the *pfcrt*-modified Dd2 lines. Download TABLE S1, PDF file, 0.1 MB. Copyright © 2017 Dhingra et al. 2017 Dhingra et al. This content is distributed under the terms of the Creative Commons Attribution 4.0 International license.

10.1128/mBio.00303-17.7 TABLE S2  Representative parasitemias of the Dd2^Dd2+C101F^ line at different piperaquine concentrations. Download TABLE S2, PDF file, 0.04 MB. Copyright © 2017 Dhingra et al. 2017 Dhingra et al. This content is distributed under the terms of the Creative Commons Attribution 4.0 International license.

As a separate assessment of PPQ susceptibility, we measured 50% lethal dose (LD_50_) values using cytocidal assays, in which parasites were exposed to a range of PPQ concentrations for 6 h. The drug was then removed by extensive washing, and parasitemia was assessed 48 h later by flow cytometry. LD_50_ values were calculated by plotting the percentage of survival against log-transformed drug concentrations, using untreated parasites as the 100% survival benchmark. We observed a 3-fold increase in the LD_50_ level in both Dd2^Dd2+C101F^ clones compared to the Dd2^Dd2^ control (*P* < 0.001; Fig. 2C and D; Table S1).

Of note, a recent report also documented a mutant FCB line that had acquired the C101F mutation in PfCRT (69), as a result of selection with the antiviral agent amantadine (AMT), which is known to generally be more potent against CQ-resistant parasites (71, 72). FCB has nearly all of the 8 PfCRT mutations present in Dd2, except for the I356T mutation (39). That report documented a 2.5-fold increase in the PPQ IC_50_ in FCB^C101F^ versus the parental FCB line (69). To further examine their PPQ response, these lines were sorbitol synchronized and tested as rings or trophozoites in 6-h cytocidal assays with a range of PPQ concentrations (see Materials and Methods). Results showed a 2- to 3-fold increase in the LD_50_ values for rings and in the LD_50_ and LD_90_ values for trophozoites in FCB^C101F^ compared with FCB. The LD_90_ value for rings showed the highest relative increase of nearly 7-fold in FCB^C101F^ compared to FCB (see Fig. S2A and S2B in the supplemental material). These data match closely our observed increase in survival in PPQ-pulsed Dd2^Dd2+C101F^ parasites.

10.1128/mBio.00303-17.2 FIG S2  *In vitro* piperaquine LD_50_, LD_90_, and the ratio of IC_50_s of AMT-selected FCB lines and *pfcrt*-modified Dd2 lines to different antimalarials. (A) PPQ LD_50_ and (B) LD_90_ values of the AMT-selected FCB^C101F^ line carrying the PfCRT^C101F^ mutation (69) and its parental FCB line. Assays (performed in duplicate on two independent occasions) were conducted with tightly synchronized 0- to 3-h postinvasion ring-stage parasites or 28- to 31-h mature trophozoite stages (Trophs). Synchronized parasites were exposed to a brief 6-h pulse of PPQ, after which the drug was removed by extensive washing. Parasites were further incubated for 66 h at 37°C, and the parasitemias were determined thereafter by SYBR green fluorescence using a 96-well plate reader. Percentages of parasite survival (estimated as the ratio of parasitemia in drug-exposed to nonexposed wells) were curve fitted against log-transformed drug concentrations to estimate the LD_50_ and LD_90_ values. Values are shown as means ± range/2. (C) Ratios of the IC_50_s (or LD_50_ values for PPQ) of the PfCRT^C101F^ mutant lines (Dd2^Dd2+C101F CL1^ and Dd2^Dd2+C101F CL2^ and FCB^C101F^) compared to their Dd2 and FCB parental lines. IC_50_ data for each drug were generated from three independent 72-h dose-response assays performed in duplicate, as described previously (93), and are shown as means ± SEM. Download FIG S2, EPS file, 2.4 MB. Copyright © 2017 Dhingra et al. 2017 Dhingra et al. This content is distributed under the terms of the Creative Commons Attribution 4.0 International license.

We also observed an enlarged DV in the Dd2^Dd2+C101F^ clones at the trophozoite and schizont asexual blood stages, compared with the isogenic Dd2^Dd2^ line (example provided in Fig. S3 in the supplemental material). A similar phenotype was observed with the FCB^C101F^ line and a BSD selected 3D7 line harboring a PfCRT^L272F^ mutation (69).

10.1128/mBio.00303-17.3 FIG S3  Morphology of the Dd2 *pfcrt*-modified and parental lines. Shown is a light microscopy representation of Giemsa-stained parasites at the ring, trophozoite, and schizont stages of parasite development. Enlarged digestive vacuoles in the Dd2^Dd2+C101F^ parasites are seen at the trophozoite stage (middle panels) and the schizont stage (bottom panels) of development compared to the parental Dd2 line and the recombinant control, Dd2^Dd2^. Download FIG S3, TIF file, 1.4 MB. Copyright © 2017 Dhingra et al. 2017 Dhingra et al. This content is distributed under the terms of the Creative Commons Attribution 4.0 International license.

To begin to investigate parasite fitness, we carried out *in vitro* growth assays as a proxy measurement by comparing the *in vitro* replication rates of both Dd2^Dd2+C101F^ clones to their isogenic control line, Dd2^Dd2^. These assays were carried out by measuring the rate of expansion of different parasite lines in one 48-h asexual blood-stage cycle after seeding them at 1% starting parasitemia (see Materials and Methods). We observed a statistically significant, nearly 2-fold growth defect in the growth rate of parasites carrying the mutant *pfcrt*^C101F^ allele (*P* < 0.01) (Table 2).

**TABLE 2:** *In vitro* replication rates of the *pfcrt*-modified lines^a^

Line	Replication rate	*P* value vs:		
		Dd2^Dd2^	Dd2	Dd2^Dd2+C101F CL1^
Dd2	8.5 ± 1.5	0.24		
Dd2^Dd2^	7.3 ± 1.5			
Dd2^Dd2+C101F CL1^	4.4 ± 1.1	0.0047	0.0012	
Dd2^Dd2+C101F CL2^	3.8 ± 1.2	0.0023	0.0012	0.38

^a^ Replication rates per 48-h generation of asexual blood-stage growth are expressed as the mean ± standard deviation (SD). Assays were performed on 6 to 7 independent occasions in duplicate. Statistical comparisons were performed using two-tailed Mann-Whitney *U* tests.

### PfCRT^C101F^ alters susceptibility to different antimalarials.

**FIG 3:**
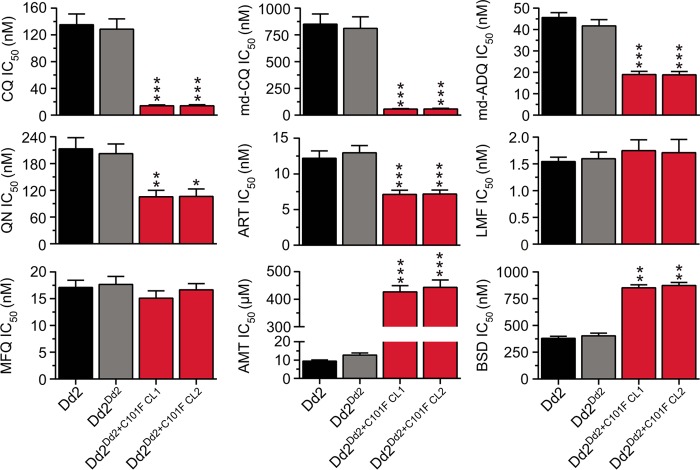
*In vitro* drug susceptibility of Dd2 recombinant lines to antimalarials. Mean ± SEM IC_50_s are shown for chloroquine (CQ), monodesethyl-chloroquine (md-CQ), monodesethyl-amodiaquine (md-ADQ), quinine (QN), artemisinin (ART), lumefantrine (LMF), mefloquine (MFQ), amantadine (AMT), and blasticidin (BSD). Data are represented in nanomolar concentration, except for those for AMT, which are expressed in micromolar concentration. (Values are listed in Table S1 in the supplemental material.) Data were obtained from 4 to 8 independent assays performed in duplicate. Statistical comparisons between Dd2^Dd2+C101F^ clones and the isogenic control Dd2^Dd2^ used two-tailed Mann-Whitney *U* tests. *, *P* < 0.05; **, *P* < 0.01; ***, *P* < 0.001.

We also assessed the *in vitro* drug susceptibility of the Dd2^Dd2+C101F^ clones to a panel of registered antimalarial drugs, using standard 72-h assays. Results showed 9-fold and 14-fold increases in susceptibility to CQ and its active metabolite md-CQ, respectively, in the Dd2^Dd2+C101F^ clones compared to parental Dd2^Dd2^ (*P* < 0.001) (Fig. 3; Table S1). This indicates that the PfCRT^C101F^ mutation, operating on the background of the Dd2 haplotype, renders parasites CQ sensitive.

Results showed a 2-fold increased susceptibility to md-ADQ (the active metabolite of the 4-aminoquinoline ADQ), quinine (QN [a cinchona alkaloid harboring a 4-aminoquinoline ring]), and ART (an endoperoxide) in the Dd2^Dd2+C101F^ clones compared to the recombinant control Dd2^Dd2^ (*P* < 0.001) (Fig. 3; Table S1). No shifts were observed in the IC_50_s of LMF or MFQ (related aminoalcohols), which are widely used ACT partner drugs. A nearly identical set of shifts in parasite susceptibility to these antimalarial agents was observed in both the Dd2^Dd2+C101F^ and the FCB^C101F^ lines (Fig. S2C).

A 35-fold increase in AMT IC_50_s was also observed in recombinant Dd2 clones harboring the C101F mutation compared to Dd2^Dd2^ (*P* < 0.01) (Fig. 3; Table S1), consistent with the appearance of this mutation in the AMT-resistant FCB^C101F^ parasites (69). Our data also documented a 2-fold increase in the BSD IC_50_s of Dd2^Dd2+C101F^ clones compared to Dd2^Dd2^ (*P* < 0.001). These findings highlight how mutant PfCRT isoforms are paradoxically selective yet pleotropic in modulating *P. falciparum* susceptibility to a wide diversity of antimalarial agents.

### PPQ inhibits Hz formation in a dose-dependent manner.

To investigate PPQ’s mode of action and the impact of the PfCRT^C101F^ mutation in more mechanistic detail, we determined the effect of different concentrations of PPQ on Hz biocrystallization using a pyridine-labeled heme fractionation assay (73, 74). This assay spectroscopically determines the mass of Hb, free heme (which can be associated with neutral lipids or other parasite biomolecules), and Hz present in trophozoites after treatment with increasing concentrations of a drug (in our case either PPQ or the reference drug CQ). Assays were performed using a range of IC_50_ or LD_50_ assays, generated in the same lab as the heme measurements using a lactate dehydrogenase-based assay (75). The measurements of heme and Hz quantify the parasite’s ability to detoxify reactive free heme through Hz formation in the presence of drug, where an increase in free heme corresponds to a decrease in parasite survival. The control amount of Hb, free heme, and Hz that was determined indicates the amount of each species that was produced and tolerated under normal culture conditions, in the absence of drug.

**FIG 4:**
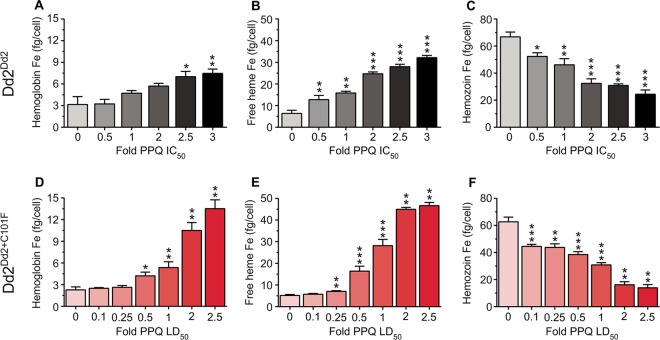
Heme fractionation of piperaquine (PPQ)-treated Dd2^Dd2^ and Dd2^Dd2+C101F^ (clone 1). (A to C) Amounts of (A) hemoglobin Fe (femtograms per cell), (B) free heme Fe (femtograms per cell), and (C) hemozoin Fe present in Dd2^Dd2^ treated with increasing concentrations of PPQ (used at multiples of the Dd2^Dd2^ PPQ IC_50_). (D to F) Amounts of (D) hemoglobin Fe, (E) free heme Fe, and (F) hemozoin Fe present at increasing concentrations of PPQ-treated Dd2^Dd2+C101F^ clone 1 (multiples of the Dd2^Dd2+C101F^ PPQ LD_50_). Data are presented as means ± SEM (calculated from 4 to 8 values per concentration). Statistical comparisons of the drug-treated parasites compared to their untreated controls were performed using two-tailed Mann-Whitney *U* tests. *, *P* < 0.05; **, *P* < 0.01; ***, *P* < 0.001.

**FIG 5:**
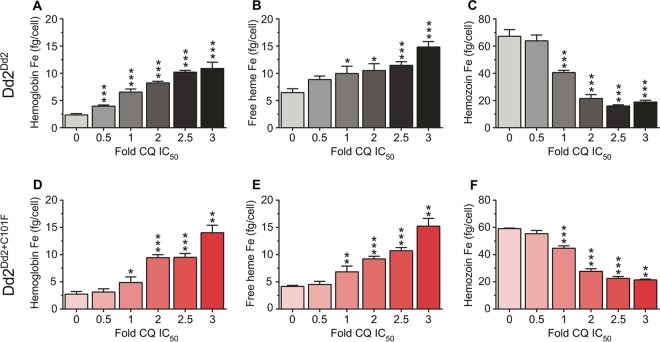
Heme fractionation of chloroquine (CQ)-treated Dd2^Dd2^ and Dd2^Dd2+C101F^ (clone 1). (A to C) Amounts of (A) hemoglobin Fe (femtograms per cell), (B) free heme Fe (femtograms per cell), and (C) hemozoin Fe (femtograms per cell) present in Dd2^Dd2^ treated with increasing concentrations of CQ (used at multiples of the Dd2^Dd2^ CQ IC_50_). (D to F) Amounts of (D) hemoglobin Fe, (E) free heme Fe, and (F) hemozoin Fe present in Dd2^Dd2+C101F^ parasites treated with multiples of their CQ IC_50_. Data (calculated from 6 to 8 values per concentration) are presented and statistically analyzed as per Fig. 4.

In the presence of high concentrations of PPQ or CQ, we observed that the parasite’s ability to catabolize Hb was compromised, as shown by the statistically significant dose-dependent increase in undigested Hb observed in drug-treated Dd2^Dd2^ and Dd2^Dd2+C101F^ (Fig. 4A and D and Fig. 5A and D; see Fig. S4 and Table S3 in the supplemental material). With PPQ, this effect was the most pronounced with the PPQ-resistant Dd2^Dd2+C101F^ line (Fig. 4A and D). In terms of drug concentrations, significant increases occurred starting in the range of 73 to 183 nM for both drugs and both parasite lines (Fig. S4). At similar concentrations, undegraded Hb levels were generally higher in the parasites that were drug-sensitive (Dd2^Dd2+C101F^ in the case of CQ and Dd2^Dd2^ in the case of PPQ).

10.1128/mBio.00303-17.4 FIG S4  Heme fractionation of PPQ- and CQ-treated Dd2^Dd2^ and Dd2^Dd2+C101F^ (clone 1). (A to C) Amounts of (A) hemoglobin Fe (femtograms per cell), (B) free heme Fe (femtograms per cell), and (C) hemozoin Fe present in Dd2^Dd2^ treated with increasing concentrations of PPQ. (D to F) Amounts of (D) hemoglobin Fe, (E) free heme Fe, and (F) hemozoin Fe present at increasing concentrations of PPQ-treated Dd2^Dd2+C101F^. (G to I) Amounts of (G) hemoglobin Fe, (H) free heme Fe, and (I) hemozoin Fe present in Dd2^Dd2^ treated with increasing concentrations of CQ. (J to L) Amounts of (J) hemoglobin Fe, (K) free heme Fe, and (L) hemozoin Fe present in Dd2^Dd2+C101F^ parasites treated with increasing concentrations of CQ. Data are presented as means ± SEM (calculated from 4 to 8 values per concentration). Statistical comparisons of the drug-treated lines to their untreated controls were performed using two-tailed Mann-Whitney *U* tests. *, *P* < 0.05; **, *P* < 0.01; ***, *P* < 0.001. Download FIG S4, EPS file, 1.9 MB. Copyright © 2017 Dhingra et al. 2017 Dhingra et al. This content is distributed under the terms of the Creative Commons Attribution 4.0 International license.

10.1128/mBio.00303-17.8 TABLE S3  Hemoglobin, free heme, and hemozoin levels in drug-treated *pfcrt*-modified Dd2 parasites. Download TABLE S3, PDF file, 0.05MB. Copyright © 2017 Dhingra et al. 2017 Dhingra et al. This content is distributed under the terms of the Creative Commons Attribution 4.0 International license.

We also examined levels of free heme, a product of Hb proteolysis that is known to be the most toxic heme-containing species in the parasite. For PPQ, Dd2^Dd2+C101F^ testing was extended to low LD_50_ multiples, because at 2× PPQ LD_50_ values we already observed exceptionally high levels of free heme, and cell recovery at higher PPQ concentrations was substantially reduced. With PPQ-treated Dd2^Dd2^ and Dd2^Dd2+C101F^, the results showed a statistically significant dose-dependent increase in free heme observed from 0.5× PPQ IC_50_ (22.2 nM) and upward in Dd2^Dd2^ and from 0.25× PPQ LD_50_ (40.6 nM) and upward in Dd2^Dd2+C101F^ (Fig. 4B and E; see Fig. S4 in the supplemental material). The largest amount of free heme Fe produced in Dd2^Dd2^ parasites exposed to PPQ (at 3× IC_50_, i.e., 133.5 nM) was 32.1 ± 1.1 fg per cell, whereas that produced in Dd2^Dd2+C101F^ was 46.7 ± 1.5 fg per cell at 2.5× LD_50_ (i.e., 406.2 nM; note that the total amount of free heme Fe is generally ~70 to 75 fg per cell; see Table S3 in the supplemental material). Indeed, PPQ-treated Dd2^Dd2+C101F^ produced significantly higher levels of free heme than PPQ-treated Dd2^Dd2^ at the equivalent IC_50_ multiples of 50% survival or inhibition (IC_50_ and LD_50_ values were used for the Dd2^Dd2^ and Dd2^Dd2+C101F^ lines, respectively; see Table S1 in the supplemental material), suggesting that the PPQ-resistant strain was able to tolerate higher levels of heme. Of note, at similar PPQ concentrations, free heme levels were considerably lower in Dd2^Dd2+C101F^ parasites compared to Dd2^Dd2^ parasites, consistent with reduced PPQ toxicity in the C101F mutant (see Table S3 in the supplemental material; e.g., compare values in range of 40 to 90 nM).

CQ-treated Dd2^Dd2^ and Dd2^Dd2+C101F^ parasites both displayed a statistically significant dose-dependent increase in free heme from 1× IC_50_ (367 and 73 nM, respectively) and upward. The largest amount of free heme produced in CQ-treated Dd2^Dd2^ (observed at 3 × IC_50_) was 14.8 ± 1.0 fg free heme Fe per cell, which essentially matched the amount (15.2 ± 1.4 fg heme Fe per cell) observed at the equivalent CQ IC_50_ in Dd2^Dd2+C101F^ (Fig. 5B and E; Fig. S4 and Table S3). At similar CQ concentrations, free heme levels were considerably lower in the resistant Dd2^Dd2^ parasites than in the sensitive Dd2^Dd2+C101F^ parasites, consistent with reduced CQ toxicity in the Dd2 parasite.

In both PPQ- and CQ-treated parasites, the observed increase in free heme corresponded to a significant dose-dependent decrease in Hz at the equivalent IC_50_ multiples (Fig. 4C and F and 5C and F). Our studies also showed that the bulk of the heme Fe in untreated parasites was sequestered in the form of Hz. These observations collectively argue for similar modes of action of CQ and PPQ and a major impact on both drugs of the PfCRT^C101F^ mutation.

### *pfmdr1* copy number does not directly impact the parasite’s susceptibility to PPQ.

**FIG 6:**
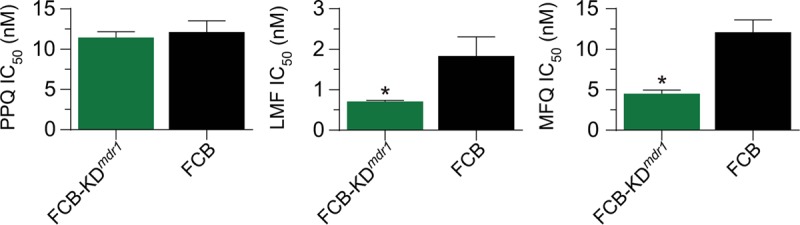
*In vitro* drug susceptibility profiles of the FCB-KD^*mdr1*^ and FCB lines. Shown are the mean ± SEM IC_50_s of the *pfmdr1* knockdown FCB-KD^*mdr1*^ and its parental FCB line to piperaquine (PPQ [left panel]), lumefantrine (LMF [middle panel]), and mefloquine (MFQ [right panel]). IC_50_s were determined in 72-h assays as per Fig. 3. Statistical comparisons of the FCB-KD^*mdr1*^ line to its nonrecombinant parental FCB line were made using two-tailed Mann-Whitney *U* tests. *, *P* < 0.05.

To examine whether *pfmdr1* copy number might play a direct role in parasite susceptibility to PPQ, we tested a pair of isogenic lines in the FCB (Southeast Asian) background, which differ in their expression levels of this gene. The FCB line expresses two copies, whereas the *pfmdr1* knockdown FCB-KD^*mdr1*^ line expresses a single copy as a result of targeted disruption of the second copy (62). Seventy-two-hour susceptibility assays revealed no difference between these two lines in their PPQ IC_50_s or dose-response profiles, which showed both lines to be fully PPQ sensitive (Fig. 6; see Table S4 in the supplemental material). As controls, we also tested LMF and MFQ in parallel, which revealed the expected 2-fold decrease in IC_50_s in the single-copy FCB-KD^*mdr1*^ line (Fig. 6; see Table S4 in the supplemental material).

10.1128/mBio.00303-17.9 TABLE S4  Mean ± SEM IC_50_ and IC_90_ values (nanomolar concentrations) of the FCB^*mdr1*-KD^ and FCB lines. Download TABLE S4, PDF file, 0.05 MB. Copyright © 2017 Dhingra et al. 2017 Dhingra et al. This content is distributed under the terms of the Creative Commons Attribution 4.0 International license.

## DISCUSSION

Here, we report that a single point mutation added to a CQ-resistant isoform of PfCRT can contribute to PPQ resistance in *P. falciparum* asexual blood-stage parasites. This mutation, C101F, earlier observed in PPQ-pressured Dd2 parasites (38), was herein introduced into parental Dd2 parasites using ZFN-based gene editing. Resistance was observed both as an ~140-fold increase in mean IC_90_ values as measured in 72-h drug susceptibility assays and as 2.5- to 3-fold increases in the LD_50_ values based on cytocidal assays that measured parasite survival after a brief (6-h) exposure to PPQ. Of note, prior studies have provided conflicting evidence about the impact of traditional CQ-resistant mutant isoforms (including Dd2, 7G8, and Cam734) on PPQ susceptibilities compared with CQ-sensitive parasites expressing wild-type PfCRT. Some reports with culture-adapted field isolates tested *in vitro* have found no difference (76, 77), a finding consistent with *in vitro* drug assay data with isogenic parasites expressing the above-mentioned mutant isoforms (78). Other data suggest an elevated PPQ IC_50_ in parasites expressing mutant *pfcrt* (26) or an increased prevalence of mutant *pfcrt* isoforms in patient cohorts following treatment with DHA+PPQ (79, 80). Our data presented herein show a significant contribution to PPQ resistance afford by the PfCRT^C101F^ mutation, which might benefit from the preexisting Dd2 mutations in our edited lines. We also document a major impact of the C101F mutation on parasite susceptibility to multiple antimalarial agents, provide evidence against a direct role for *pfmdr1* copy number in the FCB lines tested herein, and define inhibition of heme detoxification via its incorporation into Hz as the primary mode of action of this drug.

Our studies documented a nearly 2-fold reduction in replication rates in the PPQ-resistant Dd2^Dd2+C101F^ clones compared to the isogenic control line Dd2^Dd2^ (Table 2). This finding suggests that parasites harboring this mutation would be outcompeted by other, fitter parasites in patients harboring mixed infections and corroborates the absence of this mutation in a sample of ~2,500 sequenced *P. falciparum* genomes from Asia and Africa (identified using the Pf3K data available on https://www.malariagen.net/projects/pf3k) (18). By comparison, earlier studies have documented a 12 to 13% reduction in growth rate of the *pfcrt* Dd2 allele compared to that of isogenic parasites harboring wild-type *pfcrt* (78, 81). As mentioned earlier, this Dd2 allele is believed to have swept across Asia and Africa as a selective sweep driven by CQ pressure (43). Studies in high-transmission settings in Africa have shown that upon removal of CQ pressure, this allele is quite rapidly overtaken by the fitter wild-type parasites (82). As a side note, the poor growth of our C101F mutants impeded our efforts to tightly synchronize these cultures and perform PPQ survival assays, which were recently reported as an alternative method to quantify PPQ resistance *in vitro* (59). Interestingly, however, other single amino acid substitutions have been detected in PfCRT, all representing variants of the Dd2 isoform that is the most common *pfcrt* allele found in Southeast Asia (47). These mutations, namely, H97Y, M343L, and G353V, have to date only been observed in Cambodia and were recently found only in isolates that were PPQ resistant (59). Studies are under way to dissect the potential contribution of these novel PfCRT mutations to PPQ resistance in culture-adapted field isolates. These findings also emphasize the need to more closely scrutinize resistant field isolates for possible mutations in the 13-exon *pfcrt* sequence. We note that a role for novel PfCRT isoforms in PPQ resistance was also recently suggested in a study from French Guiana, which reported a rapid population-wide expansion of parasites harboring the PfCRT^C350R^ mutation, occurring on the background of the mutant 7G8 haplotype (83). Gene-edited parasites harboring this mutation introduced into the 7G8 allele, compared with isogenic parasites expressing the 7G8 isoform, displayed a slight but significant decrease in susceptibility to PPQ and had lost their CQ resistance phenotype (as observed herein with the Dd2+C101F isoform). PPQ might thus have selected for this mutation in French Guiana. An alternative explanation, which requires further experimental assessment, could be that this mutation conveys a growth advantage compared with parasites expressing the 7G8 isoform.

Results provided herein with a genetically modified FCB single-copy *pfmdr1* line and its 2-copy isogenic parent also provide evidence against *pfmdr1* copy number having any impact on PPQ susceptibility (Fig. 6), at least in this Southeast Asian background. That result leads us to conjecture that the apparent association detected in some studies between *pfmdr1* single-copy parasites and PPQ resistance in Cambodia (53–55, 58) is driven both by the removal of MFQ drug use a decade ago (67) and the occurrence and recent expansion of PPQ resistance in single-copy *pfmdr1* backgrounds. If confirmed with more recently culture-adapted parasite strains, this finding would suggest that triple ACTs that include PPQ and MFQ, currently under evaluation in clinical trials in Cambodia (84), might not benefit from an increased genetic barrier for development of resistance to both drugs.

Finally, we note that novel mutant PfCRT isoforms are likely to only be one of several factors that collectively create a multigenic basis of PPQ resistance, as evidenced by the fact that of 21 PPQ-resistant parasites recently reported (59), 13 carried variant PfCRT isoforms, whereas the other 8 harbored the PPQ-sensitive Dd2 isoform. Recent genome-wide association studies with PPQ-resistant or -sensitive field isolates have implicated increased copy number of plasmepsins 2 and 3 as markers of PPQ resistance (54, 55). One hypothesis is that these hemoglobinases could contribute to PPQ resistance by accelerating Hb degradation and reducing the intravacuolar concentration of reactive heme species that are bound by PPQ (54). Results of transfection studies to directly assess the role of the plasmepsins are keenly awaited.

Our study provides compelling evidence that PPQ acts primarily by preventing Hz formation, causing a buildup of reactive free heme, likely in a drug-bound state. To our knowledge, this is the first report documenting PPQ inhibition of Hz formation, with concentration-response profiles that closely match those of CQ. Indeed, CQ is a well-characterized heme-binding drug that like PPQ has a core 4-aminoquinoline moiety that physically intercalates with the β-hematin dimeric form of heme (85). One can envisage PPQ acting similarly, with its two 4-aminoquinoline moieties binding across two β-hematin lattices in the Hz multilayer crystal, thus preventing further extension of the crystals and resulting in accumulation of reactive, membrane-lytic nonbound heme species. Interestingly, both PPQ and CQ also inhibited Hb proteolysis, generally at higher concentrations than those that inhibited Hz formation, suggesting a secondary upstream mode of action of both drugs. A similar profile was previously observed in CQ-treated drug-sensitive D10 or 3D7 parasites (73, 86). This aspect of PPQ and CQ action merits further attention, both with trophozoites where Hb catabolism peaks and with earlier ring-stage parasites when Hb endocytosis begins (87). Concentration-dependent effects of PPQ on Hz formation and Hb proteolysis might also contribute to the bimodal PPQ resistance profiles observed in the two Dd2^Dd2+C101F^ clones (Fig. 2B).

We also note that PPQ-treated parasites also produced much higher levels of free heme than those treated with CQ at comparable drug concentrations. Our assay cannot distinguish between heme bound to drug that can exchange with pyridine and other forms of free or loosely bound heme. Further studies are required to assess how heme and heme-drug complexes differ in their ability to trigger parasite death. Nonetheless, for both drugs the resistant parasites demonstrated lower free heme levels than their sensitive counterparts at the same external drug concentrations, consistent with the resistant parasites achieving lower drug toxicity in the DV.

Radiolabeled-drug accumulation studies provide evidence that both CQ and PPQ, which act as weak-base drugs, can concentrate several orders of magnitude in the parasite, where they are predicted to accumulate as diprotonated species in the highly acidic DV (38, 88–91). As cited in the introduction, multiple lines of evidence suggest that CQ-resistant parasites reduce the DV concentration of CQ by actively effluxing this drug, in a proton-dependent manner, through mutant PfCRT isoforms such as Dd2. We posit that the introduction of the C101F mutation into the Dd2 isoform, whereby cysteine is replaced by the bulky hydrophobic amino acid residue phenylalanine in the second predicted transmembrane domain, might similarly confer upon mutant PfCRT the ability to efflux PPQ away from its site of action in the DV. PPQ transport studies, using cultured parasites or heterologous expression assays, will be useful to further examine whether this variant can transport PPQ. Of note, a recent study in a *Xenopus laevis* oocyte heterologous expression system provided evidence of how PfCRT mutations could affect drug transport kinetics (*K*_*m*_ and *V*_max_) and how these could change drug binding to transporter substrate-binding sites (92). It was suggested that this binding inhibits PfCRT’s native transport function, providing an alternative mode of drug action.

The *Xenopus* study also investigated the V369F mutation that arose in PfCRT following AMT selection of a K1 strain harboring a K76I variant that was moderately CQ resistant. Interestingly that line (harboring the same bulky phenylalanine substitution as our C101F mutant) also saw a significant reduction in CQ IC_50_s (92), as seen in our Dd2^Dd2+C101F^ line that became AMT resistant and CQ sensitive. This finding led the authors to propose that the V369F mutation ablated PfCRT-mediated CQ and AMT transport, thereby trapping both protonatable agents in the DV, where CQ acts and AMT is sequestered away from its primary site of action in the parasite cytosol. In this light, an alternative interpretation for how the C101F mutation might contribute to PPQ resistance would be that this mutation reduces PPQ binding to PfCRT, while simultaneously ablating this transporter’s ability to efflux CQ and thus rendering parasites susceptible to CQ action. PPQ drug-binding studies with the Dd2 isoform and the C101F variant would be particular interesting to further examine this proposed aspect of PPQ action.

By microscopic examination, we observed significant swelling of the DV in our Dd2^Dd2+C101F^ clones, as observed with other lines expressing PfCRT^C101F^ or PfCRT^L272F^ variants (69). Based on earlier peptidomic analysis of mutant PfCRT (81), we suspect that this “monster DV” phenotype might reflect an increased accumulation of solutes, possibly globin-derived peptides, in the DV as a result of the C101F mutation impairing PfCRT function.

Our findings also reveal a pleiotropic impact of this C101F mutation on multiple antimalarial agents, with a loss of resistance to the active metabolites of the related drugs CQ and ADQ, as well as to QN. A very similar set of changes was observed in the FCB^C101F^ line. This result is consistent with earlier transfection or drug-selection-based studies that document PfCRT as a major determinant of parasite resistance to these drugs, an effect that depends on the PfCRT isoform (40, 61, 69, 93). Our discovery here that one additional point mutation in PfCRT can confer resistance to one clinical agent while reversing resistance to others highlights the value of combining agents with opposing selective pressures to force parasites into evolutionary traps whereby dual resistance is harder to achieve (94). This idea is also supported by evidence suggesting that PfCRT itself is not just a mediator of resistance but might also be a drug target whose functional inhibition could be an important component of drug action (92, 93, 95, 96). Finally, we note that our PfCRT^C101F^ mutants also rendered parasites more susceptible to ART, presumably via an impact on the physiological state of the DV wherein the ART-activating agent heme is liberated during Hb degradation (97).

Drug resistance has long thwarted global efforts to effectively treat malaria and reduce its impact on countries where the disease is endemic. Our data illustrate that additional evolution of mutant PfCRT represents a path toward achieving PPQ resistance, presumably as one component of a multigenic trait that must balance the need for resistance with minimal fitness cost to the parasite and adequate transmissibility. Defining the genetic basis and molecular features of resistance to ACT drugs, including PPQ is urgently required as a means to identify appropriate alternatives to prevent their spread and maintain the remarkable progress made against malaria in the past 15 years (1).

## MATERIALS AND METHODS

### Plasmid construction.

The pZFN^14/15^-*bsd* and p*crt*^Dd2^-h*dhfr* plasmids have been previously described (60). pZFN^14/15^-*bsd* expresses a pair of *pfcrt* intron 1/exon 2-specific ZFNs linked via a viral 2A “ribosome skip” peptide. The DNA repair template was provided on the p*crt*^Dd2+C101F^-h*dhfr* plasmid that carries exon 1, intron 1, and exons 2 to 13 of the *pfcrt* gene (Dd2 isoform). This plasmid also harbors the C101F mutation, which was introduced via site-directed mutagenesis (QuikChange Multi SDM kit; Agilent Technologies) on p*crt*^Dd2^-h*dhfr*, using primers p1 and p2 (see Table S5 in the supplemental material). This *pfcrt* sequence is flanked by a *pfcrt* 5′ untranslated region (UTR) and a *Plasmodium berghei crt* 3′ UTR. Homology-directed repair (using *pfcrt* 5′- and 3′-UTR sequences as 5′ and 3′ regions of homology) resulted in integration of this modified *pfcrt* sequence, along with a downstream human *dhfr* marker that mediates resistance to the antimalarial agent WR99210 (Fig. 1A).

10.1128/mBio.00303-17.10 TABLE S5  List of oligonucleotides used in this study. Download TABLE S5, PDF file, 0.05 MB. Copyright © 2017 Dhingra et al. 2017 Dhingra et al. This content is distributed under the terms of the Creative Commons Attribution 4.0 International license.

### Parasite culturing and transfections.

*P. falciparum* asexual blood-stage parasites were cultured in human O^+^ red blood cells (RBCs) in RPMI 1640 with 11 mM glucose, supplemented with 2 mM l-glutamine, 25 mM HEPES, 2 g/liter sodium bicarbonate, 10 µg/ml gentamicin, 50 µM hypoxanthine, and 0.5% (wt/vol) AlbuMAXII (Thermo Fisher). Parasite cultures were maintained at 5% hematocrit at 37°C in an environment of 5% O_2_/5% CO_2_/90% N_2_. Transfections were performed by electroporating ring-stage Dd2 parasites at 5% parasitemia with 50 µg of purified circular plasmid DNA (98). Dd2 parasites were first transfected with the donor plasmid p*crt*^Dd2+C101F^-h*dhfr* and selected with 2.5 nM WR99210 (Jacobus Pharmaceuticals) to enrich for episomally transformed parasites. These parasites were then further transfected with pZFN^14/15^-*bsd* and selected with 2 µg/ml BSD (Thermo Fisher) for 6 days. We also applied 40 nM PPQ pressure for 6 days, beyond which the transfected cultures were selected only with 2.5 nM WR99210. Parasites were visible microscopically 4 to 6 weeks postelectroporation and were screened for editing (Fig. 1B). Positively edited bulk cultures were cloned via limiting dilution in 96-well plates (containing on average 0.25 parasite per well). These plates were screened for viable parasites after 21 to 24 days. Briefly, cells were stained with 100 nM MitoTracker Deep Red and 1 × SYBR green (Thermo Fisher) in 1× phosphate-buffered saline (PBS [pH 7.4]), incubated at 37°C for 30 min, and quantified on an Accuri C6 flow cytometer (Becton Dickinson) to identify the positive wells (99). Positively edited clones were expanded for DNA and phenotypic analysis.

### DNA analysis of clones.

*pfcrt* editing events were confirmed using a PCR-based approach (Fig. 1B). PCR amplification was performed on genomic DNA (gDNA) using primer pairs p3+p4 (Dd2^Dd2+C101F^, 2.5 kb; Dd2, no product), p5+p6 (Dd2^Dd2+C101F^, 1.2 kb; Dd2, 1.4 kb), and p6+p7 (Dd2^Dd2+C101F^, 0.4 kb; Dd2, 0.6 kb). Removal of introns from the edited Dd2 parasites results in slightly shorter PCR amplicons compared to the unedited Dd2. The presence of the PfCRT^C101F^ mutation in these edited lines was confirmed by sequencing cDNA using primers p8 to p10 (Fig. 1C; see Table S5 in the supplemental material).

### Whole-genome sequence analysis.

The Nextera XT kit (Illumina) was used to prepare DNA libraries from samples for whole-genome sequencing using the dual-index protocol. The libraries were run on an Illumina HiSeq 2500 in the Rapid Run mode with 100-bp paired-end reads. The reads were aligned to the *P. falciparum* 3D7 reference genome (PlasmoDB v.13.0) as described previously (100). Single nucleotide polymorphisms (SNPs) and inserts/deletions (indels) were called with the Genome Analysis Toolkit’s (GATK) HaplotypeCaller (101, 102). Variants were filtered by quality scores and sequencing bias statistics based on GATK’s default filtering parameters. SNPs were filtered out if they met any of the following criteria: quality depth (QD), <2.0; mapping quality (MQ), <50.0; Phred-scaled *P* value using Fisher’s exact test to detect strand bias (FS), >60.0; symmetric odds ratio (SOR), >4.0; *Z* score from Wilcoxon rank-sum test of alternative versus reference read mapping qualities (MQRankSum), <−12.5; and read positive rank sum (RPRS), <−8.0. Indels were filtered out if they met any of the following criteria: QD, <2.0; RPRS, <−20.0; FS, >200.0. Variants were annotated using snpeff (version 4.2) (103). Custom scripts were used to compare the variants between the Dd2 parent sequence and the clones.

### *In vitro* drug susceptibility assays.

We tested for changes in the *in vitro* susceptibility of the Dd2^Dd2+C101F^ clones to different antimalarials by comparing their IC_50_ and IC_90_ values to those of the recombinant isogenic control line Dd2^Dd2^ and the parental Dd2 line. IC_50_ and IC_90_ values were determined for PPQ, CQ, md-CQ, md-ADQ, QN, ART, LMF, MFQ, AMT, and BSD. md-CQ and md-ADQ are the *in vivo* metabolites of CQ and ADQ, respectively. To determine IC_50_s, we incubated parasites at 0.25% starting parasitemia and 1% hematocrit with a range of drug concentrations (across a range of 2-fold dilutions) at 37°C for 72 h in 96-well plates. Parasite growth in each well was assessed after 72 h using flow cytometry on an Accuri C6 cytometer with parasites stained with SYBR green I and MitoTracker Deep Red, as described previously (99). *In vitro* IC_50_ and IC_90_ values were determined by nonlinear regression analysis. Statistical comparisons of the Dd2^Dd2+C101F^ line to its recombinant control (Dd2^Dd2^) were made using two-tailed Mann-Whitney *U* tests on GraphPad Prism 6 software.

Cytocidal assays to measure LD_50_ values in Dd2^Dd2+C101F^ and Dd2^Dd2^ clones were performed as previously described (104) with slight modifications. Briefly, unsynchronized parasites, in 96-well plates, were incubated at 1% starting parasitemia and 2% hematocrit at 37°C across a range of PPQ concentrations. After 6 h, PPQ was removed using three rounds of washing with complete medium (by centrifuging plates at 750 × *g* for 3 min), and the plates were incubated at 37°C for an additional 48 h. Parasitemias were then measured in each well using flow cytometry. The percentage of parasite survival (estimated as the ratio of parasitemia in drug-exposed to nonexposed wells) was curve fitted against log-transformed drug concentrations, using GraphPad Prism 6 software. Statistical comparisons of LD_50_ values were carried out using two-tailed Mann-Whitney *U* tests.

Cytocidal assays on FCB^C101F^ and FCB parasites (69) employed parasites that were sorbitol synchronized and cultured as described in reference 93. Assays were performed in 12-well plates with 1-ml culture volumes at 0.2% starting parasitemia and 2% hematocrit. Following the 6-h drug exposure, cultures were washed as above, and cultured an additional 66 h. Plates were then frozen at −20°C, thawed, and mixed uniformly, and duplicate 100-μl aliquots were used to assess parasite density with SYBR green (105). Fluorescence was measured on a FLUOstar Omega plate reader (BMG Labtech, Inc.) with an excitation wavelength set at 485 nm and emission wavelength at 530 nm. Dose-response data were fit to a nonlinear sigmoidal regression function allowing for a variable slope (GraphPad Prism 6.0).

### Heme fractionation assays.

The PPQ and CQ drug susceptibility values used for the heme fractionation experiments were determined using the parasite lactate dehydrogenase assay (75). The heme profiles of the *pfcrt*-modified lines Dd2^Dd2^ and Dd2^Dd2+C101F^ were determined as described previously (74). Briefly, cultures were synchronized at 48-h intervals with 5% (wt/vol) sorbitol, and ring-stage parasites were exposed to drug at various multiples of their IC_50_ or LD_50_ values. Parasites were incubated in a gas environment consisting of 3% O_2_/4% CO_2_/93% N_2_. RBCs were harvested after 32 h, and the trophozoites were isolated with 0.05% (wt/vol) saponin and washed with 1× PBS (pH 7.5) to remove traces of the RBC Hb. RBCs and trophozoites were quantified in these samples using a hemocytometer and flow cytometry. The contents of the trophozoite pellet were then released by hypotonic lysis and sonication. Following centrifugation, the supernatants corresponding to membrane-soluble Hb (fraction 1 [see Fig. S5 in the supplemental material) were treated with 4% (wt/vol) SDS and 2.5% (vol/vol) pyridine. Pellets were again treated with 4% SDS, 2.5% pyridine, sonicated, and centrifuged. Supernatants corresponding to the free heme fraction (fraction 2) were then recovered. The remaining Hz pellets (fraction 3) were then solubilized in 4% SDS, 0.3 M NaOH and then neutralized with 0.3 M HCl, sonicated, and treated with 25% pyridine.

10.1128/mBio.00303-17.5 FIG S5  Schematic of the heme fractionation assay. This procedure was reported earlier (74). Download FIG S5, EPS file, 1.7 MB. Copyright © 2017 Dhingra et al. 2017 Dhingra et al. This content is distributed under the terms of the Creative Commons Attribution 4.0 International license.

The UV-visible spectrum of each heme fraction as an Fe(III) heme-pyridine complex was measured using a multiwell plate reader (Spectramax 340PC; Molecular Devices). The total amount of each heme species was quantified using a heme standard curve (74). The mass of each heme Fe species per trophozoite was calculated by dividing the total amount of each heme species by the corresponding number of parasites in that fraction as determined by flow cytometry (74). Statistical comparisons were made using two-tailed Mann-Whitney *U* tests on GraphPad Prism 6 software.
