# Supplementary figures and images for: A Variant PfCRT Isoform Can Contribute to Plasmodium falciparum Resistance to the First-Line Partner Drug Piperaquine
Source: mBio. 2017 May 9;8(3):e00303-17. doi: 10.1128/mBio.00303-17 (PMC5424201; doi:10.1128/mBio.00303-17)

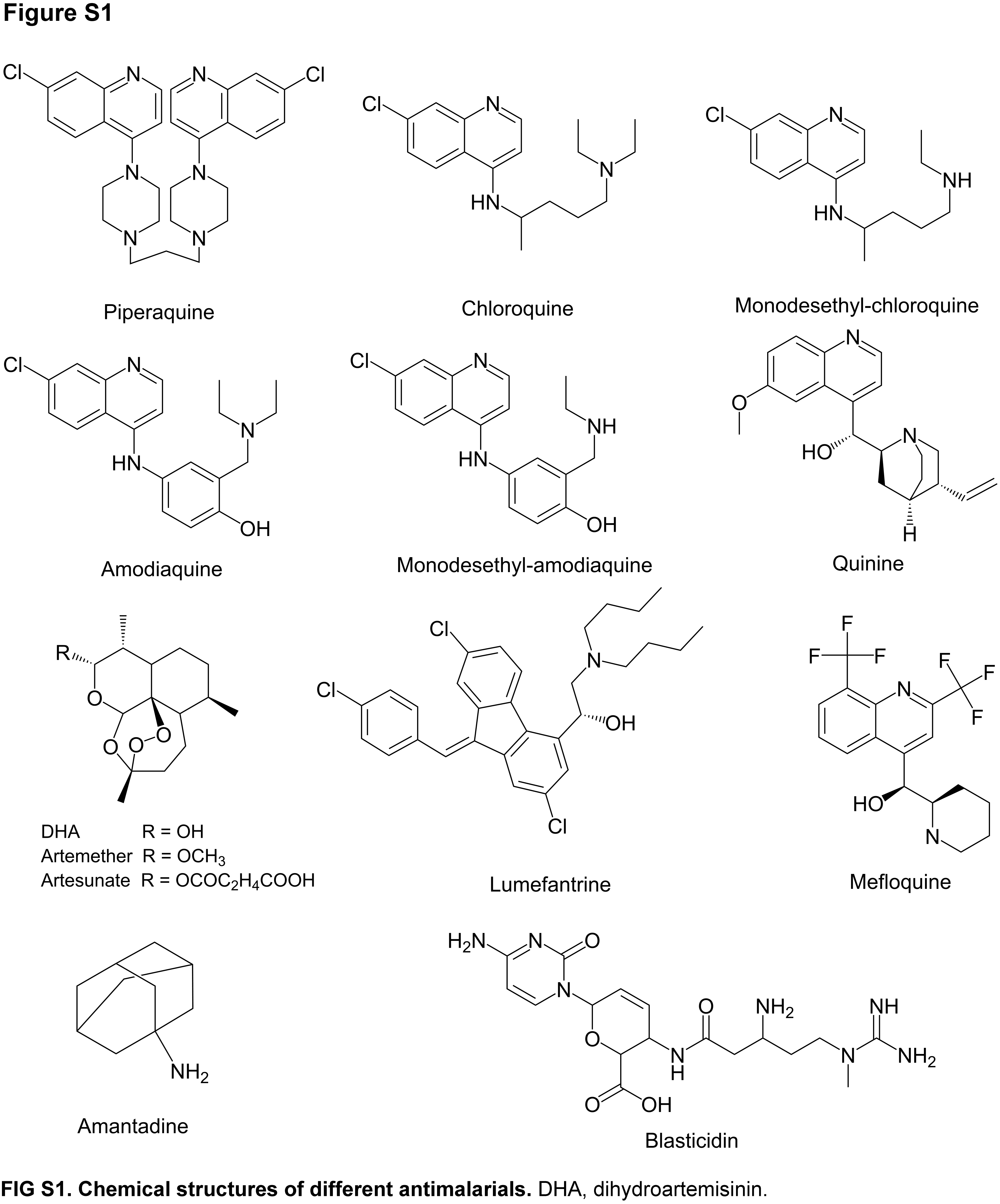

Supplement: FIG S1 [file mbo002173294sf1.tif]

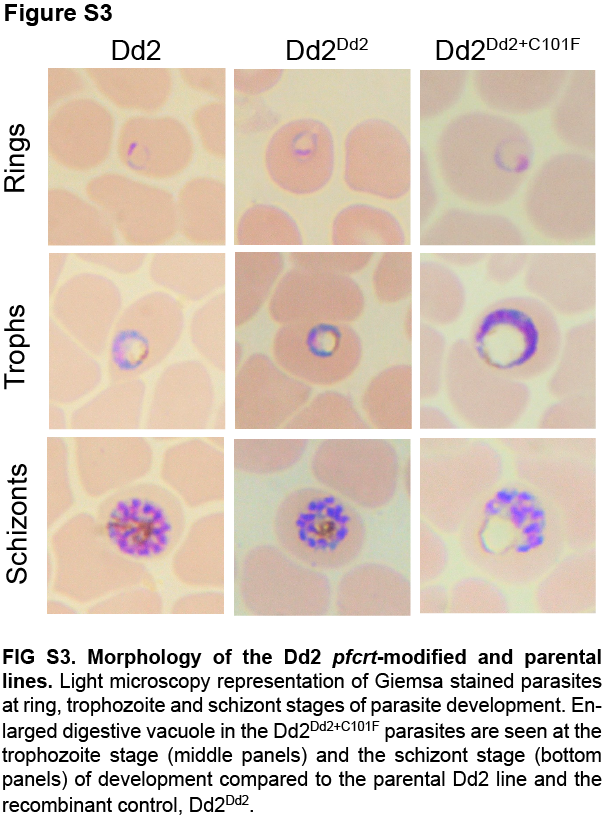

Supplement: FIG S3 [file mbo002173294sf3.tif]
