# Supplementary material for: A Variant PfCRT Isoform Can Contribute to Plasmodium falciparum Resistance to the First-Line Partner Drug Piperaquine
Source: mBio. 2017 May 9;8(3):e00303-17. doi: 10.1128/mBio.00303-17 (PMC5424201; doi:10.1128/mBio.00303-17)
Supplement: TABLE S1 [file mbo002173294st1.pdf]

TABLE S1. Mean±SEM IC_{50}, IC_{90} and LD_{50} values (nM) of the *pfcrt*-modified Dd2 lines. (page 1 of 3)

Line	Dd2	$Dd2^{Dd2}$	$Dd2^{Dd2+C101F\ CL1}$	$Dd2^{Dd2+C101F\ CL2}$
**PPQ IC_{50} (nM)**	**14.9 ± 1.3**	**15.8 ± 1.3**	**N.D.**	**N.D.**
# assays	9	9		
p value vs $Dd2^{Dd2}$	0.43			
**PPQ IC_{90} (nM)**	**27.3 ± 1.1**	**29.4 ± 1.7**	**3941.5 ± 112.6**	**4103.7 ± 141.8**
# assays	9	9	11	11
p value vs $Dd2^{Dd2}$	0.53		p<0.0001	p<0.0001
p value vs Dd2			p<0.0001	p<0.0001
p value vs $Dd2^{Dd2+C101F\ CL1}$				0.85
**PPQ LD_{50} (nM)**	**57.4 ± 7.7**	**64.7 ± 6.0**	**182.1 ± 30.9**	**163.6 ± 36.9**
# assays	7	9	7	7
p value vs $Dd2^{Dd2}$	0.34		0.0007	0.0002
p value vs Dd2			0.0012	0.0006
p value vs $Dd2^{Dd2+C101F\ CL1}$				0.45
**CQ IC_{50} (nM)**	**135.4 ± 15.9**	**128.6 ± 15.6**	**14.1 ± 1.3**	**14.2 ± 1.5**
# assays	7	7	7	7
p value vs $Dd2^{Dd2}$	0.45		0.0006	0.0006
p value vs Dd2			0.0006	0.0006
p value vs $Dd2^{Dd2+C101F\ CL1}$				0.78
**CQ IC_{90} (nM)**	**215.1 ± 13.3**	**205.1 ± 15.6**	**32.9 ± 4.4**	**32.7 ± 4.7**
# assays	7	7	7	7
p value vs $Dd2^{Dd2}$	0.38		0.0006	0.0006
p value vs Dd2			0.0006	0.0006
p value vs $Dd2^{Dd2+C101F\ CL1}$				0.87
**md-CQ IC_{50} (nM)**	**850.7 ± 95.7**	**811.5 ± 107.3**	**56.7 ± 5.5**	**58.6 ± 6.5**
# assays	7	7	7	7
p value vs $Dd2^{Dd2}$	0.78		0.0006	0.0006
p value vs Dd2			0.0006	0.0006
p value vs $Dd2^{Dd2+C101F\ CL1}$				0.60
**md-CQ IC_{90} (nM)**	**1400.0 ± 162.1**	**1396.0 ± 165.6**	**145.3 ± 18.0**	**149.4 ± 20.1**
# assays	7	7	7	7
p value vs $Dd2^{Dd2}$	0.78		0.0006	0.0006
p value vs Dd2			0.0006	0.0006
p value vs $Dd2^{Dd2+C101F\ CL1}$				0.78
**md-ADQ IC_{50} (nM)**	**45.7 ± 2.3**	**41.8 ± 2.9**	**19.0 ± 1.5**	**18.9 ± 1.6**
# assays	8	8	8	8
p value vs $Dd2^{Dd2}$	0.23		0.0002	0.0002
p value vs Dd2			0.0002	0.0002
p value vs $Dd2^{Dd2+C101F\ CL1}$				p>0.99
**md-ADQ IC_{90} (nM)**	**74.3 ± 7.6**	**67.6 ± 5.5**	**30.1 ± 1.1**	**31.9 ± 2.2**
# assays	8	8	8	8
p value vs $Dd2^{Dd2}$	0.32		0.0002	0.0002
p value vs Dd2			0.0002	0.0002
p value vs $Dd2^{Dd2+C101F\ CL1}$				0.85

TABLE S1 (cont.). Mean±SEM IC_{50}, IC_{90} and LD_{50} values (nM) of the *pfcrt*-modified Dd2 lines. (page 2 of 3)

Line	Dd2	$Dd2^{Dd2}$	$Dd2^{Dd2+C101F\ CL1}$	$Dd2^{Dd2+C101F\ CL2}$
**QN IC_{50} (nM)**	**213.2 ± 25.1**	**202.1 ± 22.1**	**105.6 ± 14.8**	**106.4 ± 16.6**
# assays	6	6	6	6
p value vs $Dd2^{Dd2}$	0.57		0.0087	0.02
p value vs Dd2			0.0087	0.02
p value vs $Dd2^{Dd2+C101F\ CL1}$				0.90
**QN IC_{90} (nM)**	**490.2 ± 39.2**	**477.6 ± 37.6**	**346.4 ± 41.4**	**343.5 ± 46.4**
# assays	6	6	6	6
p value vs $Dd2^{Dd2}$	0.79		0.06	0.09
p value vs Dd2			0.04	0.06
p value vs $Dd2^{Dd2+C101F\ CL1}$				0.90
**ART IC_{50} (nM)**	**12.2 ± 1.0**	**13.0 ± 1.0**	**7.1 ± 0.6**	**7.2 ± 0.6**
# assays	8	8	8	8
p value vs $Dd2^{Dd2}$	0.78		0.0006	0.0006
p value vs Dd2			0.0006	0.0006
p value vs $Dd2^{Dd2+C101F\ CL1}$				0.93
**ART IC_{90} (nM)**	**20.3 ± 1.4**	**21.6 ± 0.9**	**15.9 ± 1.3**	**16.1 ± 1.3**
# assays	8	8	8	8
p value vs $Dd2^{Dd2}$	0.70		0.0047	0.0047
p value vs Dd2			0.02	0.03
p value vs $Dd2^{Dd2+C101F\ CL1}$				p>0.99
**LMF IC_{50} (nM)**	**1.5 ± 0.1**	**1.6 ± 0.1**	**1.7 ± 0.2**	**1.7 ± 0.2**
# assays	7	7	7	7
p value vs $Dd2^{Dd2}$	0.87		0.87	0.87
p value vs Dd2			0.60	0.97
p value vs $Dd2^{Dd2+C101F\ CL1}$				0.78
**LMF IC_{90} (nM)**	**7.2 ± 0.7**	**7.3 ± 0.9**	**7.7 ± 0.8**	**7.3 ± 0.8**
# assays	7	7	7	7
p value vs $Dd2^{Dd2}$	0.87		0.60	0.87
p value vs Dd2			0.69	0.97
p value vs $Dd2^{Dd2+C101F\ CL1}$				0.87
**MFQ IC_{50} (nM)**	**17.1 ± 1.3**	**17.7 ± 1.5**	**15.1 ± 1.4**	**16.7 ± 1.2**
# assays	4	4	5	5
p value vs $Dd2^{Dd2}$	0.83		0.41	0.71
p value vs Dd2			0.41	0.71
p value vs $Dd2^{Dd2+C101F\ CL1}$				0.41
**MFQ IC_{90} (nM)**	**42.4 ± 1.6**	**43.1 ± 1.4**	**39.1 ± 2.5**	**42.5 ± 1.3**
# assays	4	4	5	5
p value vs $Dd2^{Dd2}$	0.66		0.29	0.71
p value vs Dd2			0.41	0.68
p value vs $Dd2^{Dd2+C101F\ CL1}$				0.15
**AMT IC_{50} (µM)**	**9.4 ± 0.7**	**12.7 ± 1.2**	**427.0 ± 22.4**	**443.2 ± 26.8**
# assays	7	6	7	7
p value vs $Dd2^{Dd2}$	0.04		0.001	0.001
p value vs Dd2			0.0006	0.0006
p value vs $Dd2^{Dd2+C101F\ CL1}$				0.60
**AMT IC_{90} (µM)**	**33.8 ± 2.2**	**63.4 ± 6.7**	**691.0 ± 74.0**	**704.5 ± 72.4**
# assays	7	6	7	7
p value vs $Dd2^{Dd2}$	0.001		0.001	0.001
p value vs Dd2			0.0006	0.0006
p value vs $Dd2^{Dd2+C101F\ CL1}$				0.45
p value vs $Dd2^{Dd2+C101F\ CL1}$				

TABLE S1 (cont). Mean±SEM IC_{50}, IC_{90} and LD_{50} values (nM) of the *pfcrt*-modified Dd2 lines. (page 3 of 3)

Line	Dd2	$Dd2^{Dd2}$	$Dd2^{Dd2+C101F\ CL1}$	$Dd2^{Dd2+C101F\ CL2}$
**BSD IC_{50} (nM)**	**381.0 ± 18.2**	**404.3 ± 24.2**	**852.4 ± 28.3**	**874.3 ± 28.5**
# assays	8	8	8	8
p value vs $Dd2^{Dd2}$	0.43		0.0002	0.0002
p value vs Dd2			0.0002	0.0002
p value vs $Dd2^{Dd2+C101F\ CL1}$				0.70
**BSD IC_{90} (nM)**	**802.4 ± 61.2**	**887.3 ± 64.6**	**1737.0 ± 112.9**	**1812.0 ± 104.5**
# assays	8	8	8	8
p value vs $Dd2^{Dd2}$	0.43		0.0002	0.0002
p value vs Dd2			0.0002	0.0002
p value vs $Dd2^{Dd2+C101F\ CL1}$				0.63

Mean±SEM LD_{50}, IC_{50} and IC_{90} values are represented in nM (except for amantadine (AMT), which is represented in µM). LD_{50}, IC_{50} and IC_{90} values were determined from 4–11 independent assays performed in duplicate. PPQ, piperaquine; CQ, chloroquine; md-CQ, monodesethyl-chloroquine; md-ADQ, monodesethyl-amodiaquine; ART, artemisinin; LMF, lumefantrine; MFQ, mefloquine; BSD, blasticidin; QN, quinine. Statistical comparisons of the recombinant C101F-edited lines to the $Dd2^{Dd2}$ control were made using two-tailed Mann-Whitney *U* tests.

Shading code:	ns	* $p<0.05$	** $p<0.01$	*** $p<0.001$
