# Supplementary material for: A Variant PfCRT Isoform Can Contribute to Plasmodium falciparum Resistance to the First-Line Partner Drug Piperaquine
Source: mBio. 2017 May 9;8(3):e00303-17. doi: 10.1128/mBio.00303-17 (PMC5424201; doi:10.1128/mBio.00303-17)
Supplement: TABLE S2 [file mbo002173294st2.pdf]

TABLE S2. Representative parasitemias of the Dd2$^{Dd2+C101F}$ line at different piperaquine concentrations.

PPQ (nM)	Dd2$^{Dd2+C101F\ CL1}$	Dd2$^{Dd2+C101F\ CL2}$	PPQ (nM)	Dd2$^{Dd2+C101F\ CL1}$	Dd2$^{Dd2+C101F\ CL2}$
500	1.53	1.31	5000	0.60	0.43
250	1.77	1.38	2500	1.55	1.15
125	1.51	1.26	1250	1.66	1.27
62.5	1.70	0.85	625	1.44	1.13
31.3	3.16	1.97	312.5	1.39	1.09
15.6	3.99	2.85	156.3	1.37	1.13
7.8	4.34	3.36	78.1	1.00	1.01
3.9	4.44	3.26	39.1	0.79	0.65
2.0	4.50	3.29	19.5	1.57	0.95
1.0	4.54	3.20	9.8	3.07	1.70
No drug control	4.37	3.24	No drug control	4.42	3.17
No drug control	4.56	3.27	No drug control	4.38	3.29

Mean parasitemias of the Dd2$^{Dd2+C101F}$ parasite lines at different piperaquine (PPQ) concentrations (2-fold dilutions), as determined from 72 h *in vitro* drug susceptibility assays. Parasitemias were determined using flow cytometry. Data represent parasitemias for 2 and 3 independent assays for drug assays starting with maximum PPQ concentrations of 500 nM and 5000 nM, respectively.
