# Supplementary material for: A Variant PfCRT Isoform Can Contribute to Plasmodium falciparum Resistance to the First-Line Partner Drug Piperaquine
Source: mBio. 2017 May 9;8(3):e00303-17. doi: 10.1128/mBio.00303-17 (PMC5424201; doi:10.1128/mBio.00303-17)
Supplement: TABLE S3 [file mbo002173294st3.pdf]

TABLE S3. Hemoglobin, free heme and hemozoin levels in drug-treated *pfcrt*-modified Dd2 parasites.

	$Dd2^{Dd2}$				$Dd2^{Dd2+C101F}$		
**PPQ drug treatment**	**Amount of hemoglobin (fg/cell)**	**Amount of free heme (fg/cell)**	**Amount of hemozoin (fg/cell)**	**PPQ drug treatment**	**Amount of hemoglobin (fg/cell)**	**Amount of free heme (fg/cell)**	**Amount of hemozoin (fg/cell)**
No drug control	3.2 ± 1.1	6.4 ± 1.5	66.8 ± 3.6	No drug control	2.3 ± 0.4	5.2 ± 0.5	62.7 ± 3.5
--	--	--	--	0.1x LD_{50} (16.2 nM)	2.5 ± 0.1	5.7 ± 0.4	44.6 ± 1.4***
--	--	--	--	0.25x LD_{50} (40.6 nM)	2.6 ± 0.2	7.1 ± 0.4**	43.7 ± 2.7**
0.5x IC_{50} (22.2 nM)	3.2 ± 0.6	12.8 ± 1.9**	52.4 ± 2.7*	0.5x LD_{50} (81.2 nM)	4.2 ± 0.5*	16.4 ± 2.3***	38.5 ± 2.2***
1.0x IC_{50} (44.5 nM)	4.7 ± 0.4	15.9 ± 0.8**	46.2 ± 4.5**	1.0x LD_{50} (162.5 nM)	5.4 ± 0.8**	28.2 ± 2.9***	30.9 ± 1.7***
2.0x IC_{50} (89.0 nM)	5.7 ± 0.4	24.7 ± 0.8***	32.5 ± 3.4***	2.0x LD_{50} (325.0 nM)	10.5 ± 1.1**	45.0 ± 0.9**	16.1 ± 2.3**
2.5x IC_{50} (111.2 nM)	7.0 ± 0.7*	28.0 ± 1.1***	30.9 ± 1.2***	2.5x LD_{50} (406.2 nM)	13.5 ± 1.2**	46.7 ± 1.5**	14.0 ± 2.4***
3.0x IC_{50} (133.5 nM)	7.5 ± 0.6**	32.1 ± 1.1***	24.4 ± 3.2***	--	--	--	--

	$Dd2^{Dd2}$				$Dd2^{Dd2+C101F}$		
**CQ drug treatment**	**Amount of hemoglobin (fg/cell)**	**Amount of free heme (fg/cell)**	**Amount of hemozoin (fg/cell)**	**CQ drug treatment**	**Amount of hemoglobin (fg/cell)**	**Amount of free heme (fg/cell)**	**Amount of hemozoin (fg/cell)**
No drug control	2.4 ± 0.2	6.5 ± 0.7	67.3 ± 4.9	No drug control	2.7 ± 0.5	4.2 ± 0.2	59.1 ± 0.5
0.5x IC_{50} (183.3 nM)	4.0 ± 0.2***	8.9 ± 0.7	63.9 ± 4.3	0.5x IC_{50} (36.3 nM)	3.1 ± 0.6	4.5 ± 0.6	55.4 ± 2.4
1.0x IC_{50} (366.7 nM)	6.6 ± 0.6***	10.0 ± 1.3*	40.5 ± 1.6***	1.0x IC_{50} (72.7 nM)	4.9 ± 1.0*	6.9 ± 1.0**	44.7 ± 1.8***
2.0x IC_{50} (733.4 nM)	8.2 ± 0.3***	10.5 ± 1.3*	21.5 ± 2.8***	2.0x IC_{50} (145.4 nM)	9.4 ± 0.6***	9.2 ± 0.5***	27.7 ± 1.9***
2.5x IC_{50} (916.7 nM)	10.2 ± 0.3***	11.5 ± 0.7***	16.1 ± 0.8***	2.5x IC_{50} (181.7 nM)	9.5 ± 0.7***	10.7 ± 0.6***	22.6 ± 1.3***
3.0x IC_{50} (1100.1 nM)	10.9 ± 1.2***	14.8 ± 1.0***	18.9 ± 1.4***	3.0x IC_{50} (218.1 nM)	14.0 ± 1.4**	15.2 ± 1.4**	21.4 ± 0.6**

Mean±SEM amount of hemoglobin, free heme and hemozoin are represented as fg/cell. The amounts of heme in different parasite lines were determined by the heme fractionation assay (see methods). Parasites were treated with increasing concentrations of PPQ or CQ (at different multiples of their IC_{50} or LD_{50} values) and hemoglobin, free heme and hemozoin amounts were measured 32 h later. Data were calculated from 6–8 values per concentration. Statistical comparisons of the drug-treated lines to their untreated controls were performed using two-tailed Mann-Whitney *U* tests. *$p<0.05$; **$p<0.01$; ***$p<0.001$.
