# Supplementary material for: A Variant PfCRT Isoform Can Contribute to Plasmodium falciparum Resistance to the First-Line Partner Drug Piperaquine
Source: mBio. 2017 May 9;8(3):e00303-17. doi: 10.1128/mBio.00303-17 (PMC5424201; doi:10.1128/mBio.00303-17)
Supplement: TABLE S4 [file mbo002173294st4.pdf]

TABLE S4. Mean±SEM IC_{50} and IC_{90} values (nM) of the FCB$^{mdr1\text{-KD}}$ and FCB line.

Line	FCB	FCB$^{mdr1\text{-KD}}$
**PPQ IC_{50} (nM)**	**12.1 ± 1.4**	**11.5 ± 0.7**
# assays	4	4
p value vs FCB		0.48
**PPQ IC_{90} (nM)**	**24.2 ± 3.4**	**22.9 ± 2.6**
# assays	4	4
p value vs FCB		0.83
**LMF IC_{50} (nM)**	**1.8 ± 0.5**	**0.7 ± 0.03**
# assays	4	4
p value vs FCB		0.03
**LMF IC_{90} (nM)**	**5.6 ± 0.1**	**3.2 ± 0.2**
# assays	4	4
p value vs FCB		0.03
**MFQ IC_{50} (nM)**	**12.1 ± 1.6**	**4.5 ± 0.5**
# assays	4	4
p value vs FCB		0.03
**MFQ IC_{90} (nM)**	**28.2 ± 3.6**	**15.2 ± 2.0**
# assays	4	4
p value vs FCB		0.03

Mean±SEM IC_{50} and IC_{90} values are represented in nM. These values were determined from 4 independent assays performed in duplicate. PPQ, piperaquine; LMF, lumefantrine; MFQ, mefloquine. Statistical comparisons of the recombinant C101F-edited lines to the Dd2^{Dd2} control were made using two-tailed Mann-Whitney *U* tests.

Shading code:	ns	*p<0.05
