# Supplementary material for: A Variant PfCRT Isoform Can Contribute to Plasmodium falciparum Resistance to the First-Line Partner Drug Piperaquine
Source: mBio. 2017 May 9;8(3):e00303-17. doi: 10.1128/mBio.00303-17 (PMC5424201; doi:10.1128/mBio.00303-17)
Supplement: TABLE S5 [file mbo002173294st5.pdf]

TABLE S5. List of oligonucleotides used in this study.

Name	Nucleotide Sequence (5'–3')	Description	Lab Name
p1	CCGAAACTCACAACTTTATT**TTT**ATGATTATGTTC	SDM C101F Fw	p3802
p2	GAACATAATCAT**AAA**AATAAAGTTGTGAGTTTCGG	SDM C101F Rw	p3803
p3	CTCGAGATGGTTGGTTCGCTAAACTGC	h*dhfr* (+1-21) Xho1 Fw	p3315
p4	TTGACCCTTATATATTCCACCCA	*Pfcrt* 3' UTR (+1285-1308)	p3403
p5	CTTGGGCCCAAGTTGTACTGCTTCTAAGC	*Pfcrt* 5' UTR (-494-517) ApaI Fw	p3404
p6	CTTATCGATAAGCAGAAGAACATATTAATAGGAATACTTAATTG	*Pfcrt* exon 3 ClaI Rv	p3265
p7	CTTGAATTCGACCTTAACAGATGGCTCAC	*Pfcrt* exon 2 EcoRI Fw	p3264
p8	CCGTTAATAATAAATACACGCAG	*Pfcrt* 5' UTR Fw	p4396
p9	GTGACATTTACAAAAATCATTTCCATG	*Pbcrt* 3' UTR Rv	p4413
p10	TCAAACATGACAAGGGAAATAGT	*Pfcrt* exon 5 Fw	p2427

Red, bold and underlined nucleotides represent SNPs incorporated into the primer sequences for site-directed mutagenesis.
